# Isolation, Characterization, and Selection of Molds Associated to Fermented Black Table Olives

**DOI:** 10.3389/fmicb.2017.01356

**Published:** 2017-07-18

**Authors:** Simona L. Bavaro, Antonia Susca, Jens C. Frisvad, Maria Tufariello, Agathi Chytiri, Giancarlo Perrone, Giovanni Mita, Antonio F. Logrieco, Gianluca Bleve

**Affiliations:** ^1^Consiglio Nazionale delle Ricerche—Istituto di Scienze delle Produzioni Alimentari Bari, Italy; ^2^Department of Biotechnology and Biomedicine, Technical University of Denmark Kongens Lyngby, Denmark; ^3^Consiglio Nazionale delle Ricerche—Istituto di Scienze delle Produzioni Alimentari Lecce, Italy; ^4^Section of Food Chemistry, Department of Chemistry, University of Ioannina Ioannina, Greece

**Keywords:** table olives, fermentation, molds, starter, mycotoxins

## Abstract

Table olives are one of the most important fermented food in the Mediterranean countries. Apart from lactic acid bacteria and yeasts that mainly conduct the olive fermentation, molds can develop on the brine surface, and can have either deleterious or useful effects on this process. From the food safety point of view, occurring molds could also produce mycotoxins, so, it is important to monitor and control them. In this respect, identification of molds associated to two Italian and two Greek fermented black table olives cultivars, was carried out. Sixty strains were isolated and molecularly identified as *Penicillium crustosum* (21), *P. roqueforti* (29), *P. paneum* (1), *P. expansum* (6), *P. polonicum* (2), *P. commune* (1). A group of 20 selected isolates was subjected to technological (beta-glucosidase, cellulolytic, ligninolytic, pectolytic, and xylanolytic activities; proteolytic enzymes) and safety (biogenic amines and secondary metabolites, including mycotoxins) characterization. Combining both technological (presence of desired and absence of undesired enzymatic activities) and safety aspects (no or low production of biogenic amines and regulated mycotoxins), it was possible to select six strains with biotechnological interest. These are putative candidates for future studies as autochthonous co-starters with yeasts and lactic acid bacteria for black table olive production.

## Introduction

During fermentation of table olives, molds can develop on the brine surface and produce a thick layer on the top. Mold growth during storage in the market can result in appearance of visible mycelia. They are generally considered spoilage microorganisms responsible for product alterations, such as flesh softening and development of moldy taste, flavor, and appearance. In table olives, the most representative identified mold genera are *Aspergillus* and *Penicillium* (Fernandez et al., 1997). Their presence reduces product acceptance by the consumers, and it is also of relevant interest for the safety of table olives, since they can be responsible for mycotoxin production. The occurrence of *Penicillium citrinum* and *P. verrucosum* during fermentation, in particular in black olives, was linked to the production of ochratoxin A (OTA) and citrinin, while the contamination by aflatoxin B_1_ (AFB) is mainly related to *Aspergillus flavus* on damaged olives during drying and storage (El Adlouni et al., 2006; Ghitakou et al., 2006; Heperkan et al., 2006, 2009).

A survey, performed in Turkey in 2000–2001, for the presence of molds in several commercial products revealed that 77% of table olives examined samples contained high amount of citrinin (Heperkan et al., 2006), whereas AFB was detected in all 30 samples of table olives and olive pasta from Athens market and OTA in two out of 30 samples. In black olives from the Marmara Region (Turkey), in addition to citrinin, aflatoxin, patulin, and penicillic acid were also detected (Korukluoğlu et al., 2000). Although, their concentrations are very low, in Greek style black table olives produced in Morocco, OTA was detected in seven of ten samples, five samples contained OTA and citrinin, whereas four out of ten samples contained AFB (El Adlouni et al., 2006). In another study, although their levels were very low, AFB was found in 4 loose and in 6 packed olive samples out of 40 commercial samples, whereas OTA was found in 12 loose and in 11 packed olive samples out of 40 samples (Franzetti et al., 2011).

Even if mycotoxin levels detected in table olives were too low to cause diseases (Medina-Pradas and Arroyo-López, 2015), green and black table olives could be a possible source of mycotoxins.

Thus, some measures have been suggested to reduce the presence of molds and mycotoxin production along the entire table olive chain by applying good handling procedure during harvest, controlling storage conditions (temperature, packaging, salinity).

On the other hand, molds can be also useful microorganisms in food production. On sausage surfaces they can lead to desirable effects mainly related to successful production or consumer appeal. Selection and production of industrialized mold starter cultures allow olive producers to reduce the risks for consumer safety and to improve sausage organoleptic traits and taste. In fact, molds can have a role in sausage maturation, in aroma and texture improvement, in shortening ripening time and/or shelf life expansion. They can produce enzymes responsible of lipids and proteins modification and degradation and they can help in reduction of lipid oxidation (Sunesen and Stahnke, 2003).

In this paper, the identification of molds associated to two Italian and two Greek fermented black table olives cultivars, was carried out. For the first time, a multi-step selection protocol, consisting in both analytical and biochemical tests, has been proposed in order to identify mold candidates to be tested as autochthonous co-starter (together with already selected yeasts and lactic acid bacteria) for black table olive production.

## Materials and methods

### Brine samples

Brine samples were collected during season 2012–2013 and 2013–2014 from Italian (Apulia) and Greek (Epirus) lab-scale (Bleve et al., 2014, 2015) and industrial fermentations of Cellina di Nardò, Leccino, Kalamàta, and Conservolea table olives.

### Isolation of fungi

The isolation of fungi from brines was carried out by serial dilution of samples in sterile water solution with 0.01% Tween 80 (Sigma-Aldrich, Darmstadt, Germany) added to assist the dispersal of conidia and transferring them to agar. One hundred microliter were spread on Dichloran Rose Bengal Chlorotetracycline (DRBC; Oxoid Ltd., Hampshire, UK) agar medium (King et al., 1979), in 90-mm petri dishes in triplicate and incubated at 25°C for 5–7 days in the dark. After incubation, single-spore isolations were made according to Crous et al. (2009) for representative colonies on the basis of their morphological traits (shape, size, and color of the colony; shape of cells). All strains isolated in this study were deposited in the ITEM collection (ITEM collection: http://www.ispa.cnr.it/Collection/). In addition, a subset of representative strains of *Penicillium* subgenus *Penicillium* used by Frisvad and Samson (2004) and available in the ITEM collection were included in the analysis as reference strains for species identification.

### Molecular identification of fungi

DNA was extracted from mycelium of isolates grown in Wickerham's medium (glucose, 40 g; peptone, 5 g; yeast extract, 3 g; malt extract, 3 g; and distilled water to 1 L) and incubated in an orbital shaker (150 rpm) for 48 h at 25°C. All components of Wickerham's medium were purchased by LabM Limited (Lancashire, UK). Following incubation, the mycelia were filtered and lyophilized for total DNA extraction. DNA was extracted starting from 10 mg of lyophilized mycelium, grinded with 5 mm iron bead in Mixer Mill MM 400 (Retsch, Germany), and processed with “Wizard® Magnetic DNA Purification System for Food” kit (Promega, Madison, WI, USA). The quality of genomic DNA was determined by electrophoresis and the quantification using a Spectrophotometer ND-1000 (Thermo Fisher, Waltham, MA, USA). Isolates recovered from DRBC agar medium were sequenced firstly in ITS region and only *fungal* spp. isolates were further sequenced in beta-tubulin gene. Amplification of the ITS and β-tubulin DNA regions was performed using primers ITS4/ITS5 and Bt2a/Bt2b, respectively, specific to filamentous ascomycetes (White et al., 1990; Glass and Donaldson, 1995), according to published protocols. PCR reactions were performed in 20 μL reaction mixtures containing 1 μL DNA template (20 ng/μL), 2 μL PCR buffer, 15.3 μL ultra pure sterile water, 0.4 μL dNTP (10 mM), 0.6 μL of each primer (10 pmol/μL), and 0.1 μL Hot Master Taq DNA Polymerase (2.5 U/μL, 5 *PRIME* GmbH, Germany). Amplifications were performed in a GeneAmp PCR system 9700 (AB Applied Biosystems, CA).

PCR amplicons were purified using the enzymatic mixture EXO/SAP (Exonuclease I, *Escherichia coli*/Shrimp Alkaline Phosphatase; Thermo Fisher Scientific, Waltham, MA, USA) and sequenced in both strands using standard conditions with BigDye™ Terminator v3.0 Ready reaction Kit (Applied Biosystems, Foster City, CA, USA). Sequence reactions were analyzed using an ABI- Prism model 3730 Genetic Analyzer (Applied Biosystems, Foster City, CA, USA) after purification by gel filtration through Sephadex G-50 (Little Chalfont, UK).

Evolutionary analyses were conducted in MEGA5 (Tamura et al., 2011) and inferred using the UPGMA method (Sneath and Sokal, 1973). The percentage of replicate trees in which the associated taxa clustered together in the bootstrap test (1,000 replicates) are shown next to the branches (Felsenstein, 1985). The evolutionary distances were computed using the number of differences method (Nei and Kumar, 2000) and are in the units of the number of base differences per sequence.

### Biochemical analyses

All chemicals for biochemical analyses were purchased from Sigma-Aldrich (Darmstadt, Germany). To perform the screening tests on the biochemical activity, *Penicillium* isolates were cultivated on basal medium (LBM) containing per liter: KH_2_PO_4_1 g, ammonium tartrate 0.5 g, MgSO_4_ 7H_2_O 0.01 g, CaCl_2_ 2H_2_O 0.01 g, yeast extract 0.001 g; CuSO_4_ 5H_2_O 0.001 g; Fe_2_(SO_4_)_3_ 0.001 g; and MnSO_4_ 0.001 g, and incubated in the dark at 25°C for 5 days. After this period, agar disks (6 mm in diameter) of active mycelia were plated on solid media containing the different substrates for the detection of beta-glucosidase, lypolitic activities, cellulase, laccase, tyrosinase, xylanase, lignin modifying, pectolytic, protease (gelatin and milk agar) activities. For all enzyme activities a score 3 (intense brown), 2 (light brown), 1 (yellow-milky), 0 (white) was assigned.

#### Beta-glucosidase activity

The activity of β-glucosidase was detected by growing the test fungus on agar containing esculin (6,7- dihydroxycomarin-6-glucosidase) as the sole carbon source. Cellulolysis Basal Medium (CBM; C_4_H_12_N_2_O_6_ 5 g, yeast extract 0.1 g, KH_3_PO_4_ 1 g, MgSO_4_·7H_2_O 0.5 g, CaCl_2_·2H_2_O 0.001 g, in 1 l distilled water) was supplemented with 0.5% esculin (w/v), 1.8% (w/v) agar and autoclaved. One ml of a sterile 2% (w/v) aqueous ferric sulfate solution was aseptically added for each 100 ml of CBM. The medium was dispensed into Petri dishes, allowed to solidify, inoculated and incubated at 25°C for 7 days in darkness. A black color developed in the medium by the colonies producing β-glucosidase (Pointing et al., 1999).

#### Hemicellulolytic (xylanolytic) enzyme assays

This enzymatic activity was detected through use of the XBM medium (C_4_H_12_O_6_ 0.5 g, KH_2_PO_4_ 1 g, MgSO_4_·7H_2_O 0.5 g, yeast extract 0.1 g, CaCl_2_·2H_2_O 0.001 g in 1 L distilled water) with 4% (w/v) xylan and 1.6% (w/v) agar and autoclaved. The medium was inoculated with the test fungus and incubated at 22°C for 7 days in darkness. The plates were stained with iodine (0.25% w/v aqueous I2 and KI), xylan degradation around the colonies appeared as a yellow-opaque area against a blue/red dish purple color for under grads xylan indicated endoxylanase activity (Pointing et al., 1999).

#### Proteolytic plate assay

The extracellular proteases were detected on agar plates, using different substrates Milk Agar (Tryptone 5 g, yeast extract 2.5 g, Dextrose 1.0 g, Skim Milk powder 1 g, 1.5% agar) supplemented with 0.0015% Bromocresol green (BCG) reagent and MEA supplemented with 1% of gelatin (MEAG) at 25°C and in presence or absence of 2.5% of NaCl. On Milk Agar, the enzyme activity was detected as clearer areas surrounding the colony, indicating that hydrolysis of the substrate had occurred. Also, it was developed a method to detect proteolytic activity using MEA as basal medium supplemented with 1% of gelatin. In this case, the detection of extracellular proteases was done after staining with Coomassie Blue (0.25% w/v) in methanol–acetic acid–water (5:1:4 v/v/v) for 1 h at room temperature and destining with methanol–acetic acid (Vermelho et al., 1996). Enzyme activity was detected as clear regions surrounding the colony, indicating that hydrolysis of the substrate had occurred (Ludemann et al., 2004).

#### Pectinolytic activity

The extracellular pectinolytic activity was assessed by medium contained 500 mL of mineral salt solution, 1 g yeast extract, 15 g of agar, 5 g of pectin, and 500 mL of distilled water. The mineral salts solution contained per liter: (NH_4_)_2_SO_4_ 2 g, KH_2_ PO_4_ 4 g, Na_2_ HPO_4_ 6 g, FeSO_4_. 7 H_2_ O 0.2 g, CaCl_2_ 1 mg, H_3_BO_3_ l g, MnSO_4_ l g, ZnSO_4_ l g, CuSO_4_ l g, MoO_3_ l g, pH 7, or pH 5 as needed. This medium at pH 7 was used to detect pectate lyase production. For all tests, plates were incubated for 5–10 days and then flooded with 1% aqueous solution of hexadecyltrimethyl ammonium bromide. This reagent precipitates intact pectin in the medium and thus a clear zone around a colony in an otherwise opaque medium indicates degradation of the pectin (Hankin et al., 1975).

#### Cellulase activity

The hydrolysis of cellulose into sugars was investigated using carboxymethylcellulose (CMC) plates. CBM medium was supplemented with 2% low viscosity CMC and 1.6% agar. The plates were incubated for 5–10 days in darkness at 25°C. When the colony diameters were ~30 mm were flooded with 2% aqueous solution of Congo Red and leaved for 15 min. Removed the stain and washed the agar surface with distilled water, the plates were flood with 1 M NaCl and discolored for 15 min. The CMC degradation around the colonies appeared as yellow-opaque area against a red color for under graded CMC (Pointing et al., 1999).

#### Laccase assay

Laccase activity was determined with 2,2′-azino-di-(3-ethylbenzothialozin-6-sulfonic acid) (ABTS) as the substrate. LBM medium was supplemented with 0.1% (w/v) glucose, 1.6% (w/v) agar and sterilized. Aseptically was added 1 mL of a sterilized solution of aqueous glucose 20% to each 100 mL of growth medium prepared. The medium was inoculated with the test fungus. The plates were incubated for 10 days in darkness at 25°C. The production of laccase was detected as the formation of green color in the growth medium (Pointing et al., 1999).

#### Tyrosinase assay

The tyrosinase enzyme is implicated in the detoxification of lignin breakdown products (Eaton and Hale, 1993). The production of tyrosinase can be assayed by the well test procedure using p-cresol (4-methoxyphenol). LBM medium was supplemented with 1.6% (w/v) agar and sterilized. Aseptically was added 1 mL of a separately sterilized 20% (w/v) aqueous glucose solution to each 100 mL of growth medium prepared. Test microorganism were inoculated and incubated at 25°C in darkness for 5–10 days. Spot tests were carried out as follows. Wells of approximately 5 mm in diameter were done in the agar medium and few drops of 0.1% (w/v) p-cresol in 0.05% (w/v) aqueous glycine solution were added inside them. Presence of a red-brown color around the well indicated a positive result (Pointing et al., 1999).

#### Lignin modifying enzymes

Decolorization of the Remazol Brilliant Blue R (RBBR) by fungi has been positively correlated with production of the polyphenol oxidases lignin peroxidase, Mn-dependent peroxidase (Boominathan and Reddy, 1992) and laccase (Pointing et al., 1999). The test foresees the use of LBM medium supplemented with 0.05% (w/v) RBBR and 1.6% (w/v) agar and sterilized. Aseptically was added 1 ml of a separately sterilized 20% (w/v) aqueous glucose solution to each 100 mL of growth medium prepared. The fungi were inoculated and incubated at 25°C in darkness and examined plates daily for 10 days.

#### Production of biogenic amines

To assess the ability of the colonies to decarboxylate aminoacids producing biogenic amines a specific media has been designed. About 0.1 g of glucose, 0.06 g of bromocresol purple, 1.5% (w/v) agar, and 10 g of each amino acid to be tested were dissolved in 900 mL of demineralized water. After sterilization, 100 mL of yeast nitrogen base (Difco Laboratories, Franklin Lakes NJ, USA) solution (6.7% w/v), previously sterilized by filtration, were aseptically added. Final pH was adjusted to 5.3 ± 0.02 using HCl. The amino acids tested were histidine, phenylalanine, tyrosine, ornithine, and lysine (Sigma-Aldrich, Darmstadt, Germany). The colonies were streaked on the surface of the agar plates and then incubated at 25°C for 4 d. At the pH of the plates the dye was yellow. Slight increase of pH turned this color to purple. The reaction was considered positive if a violet halo surrounded the colonies (Gardini et al., 2006).

#### Lipolytic activity

For assaying total lipolytic activity olive oil and rhodamine B were used. Rhodamine B (1 mg/mL) was dissolved in distilled water and sterilized by filtration. Growth medium contained per liter: nutrient broth, 8 g; sodium chloride, 4 g; and agar, 10 g. The medium was adjusted to pH 7.0, autoclaved, and cooled to about 60°C. Then 2.5% (w/v) olive oil and 0.001% (w/v) of rhodamine B solution were added with vigorous stirring and emulsified by mixing for 1 min. After the medium was allowed to stand for 10 min at 60°C to reduce foaming, 20 mL of medium was poured into each plastic petri dish. Plugs of *Penicillium*, previously cultivated on LBM for 5 days, were transferred on the surface of lipolytic agar medium. Subsequently, the plates were incubated at 25°C for 48 h. Lipase activity was identified on the plate as an orange fluorescent halo around the colonies.

### Secondary metabolite profile determination

#### Growth media and conditions

*Penicillium* isolates were grown on two different media: Czapek yeast autolysate (CYA) agar (BD Biosciences, San Jose, CA, USA), and CYA agar modified with 5% NaCl and by having pH 5.5. All isolates were incubated in triplicates in both media for 12 days in darkness at 25°C. Five agar plugs (diameter 6 mm) were cut out of the colony from the center and in a radius toward the edge of the colony for the extraction of secondary metabolites was based on a standard method for cultures grown on solid medium.

#### Chemicals

LC—MS and analytical grade chemicals (Sigma-Aldrich, Darmstadt, Germany) were used. ESI—TOF tune mix was from (Agilent Technologies CA, USA). Approximately 1,500 mycotoxins and microbial metabolites used as reference standards derived from other studies (Frisvad and Thrane, 1987; Nielsen and Smedsgaard, 2003), commercial sources and from other research groups. Other standards were obtained by Sigma-Aldrich, Cayman (Ann Arbor, MI), Calbiochem, (San Diego, CA), and ICN (Irvine, CA). TebuBio (Le-Perray-en-Yvelines, France), Axxora (Bingham, UK), Biopure (Tulln, Austria). All standards were tested for original UV—VIS data, accurate mass, and relative RT from previous studies (Frisvad and Thrane, 1987). Agar plugs containing *Penicillium* colonies were extracted using a (3:2:1) (ethyl acetate:dichloromethane:methanol) mixture (Smedsgaard, 1997).

#### Fungal metabolites analysis (UHPLC—DAD—QTOFMS)

A UHPLC—DAD—QTOF method was set up for screening, with typical injection volumes of 0.1–2 μL extract. All chemicals were purchased from Sigma-Aldrich (Darmstadt, Germany). Separation was performed on a Dionex Ultimate 3,000 UHPLC system (Thermo Scientific, Dionex, CA, USA) equipped with a 100 × 2.1 mm, 2.6 μm, Kinetex C 18 column, held at a temperature of 40°C, and using a linear gradient system composed of A: 20 mmol L^−1^ formic acid in water, and B: 20 mmol L^−1^ formic acid in acetonitrile. The flow was 0.4 mL min^−1^, 90% A graduating to 100% B in 10 min, 100% B 10–13 min, and 90% A 13.1–15 min. Time-of-flight detection was performed using a maXis 3G QTOF orthogonal mass spectrometer (Bruker Daltonics, Bremen, Germany) operated at a resolving power of ~50,000 full width at half maximum (FWHM). The instrument was equipped with an orthogonal electrospray ionization source, and mass spectra were recorded in the range m/z 100–1,000 as centroid spectra, with five scans per second. For calibration, 1 μL 10 mmol L^−1^ sodium formiate was injected at the beginning of each chromatographic run, using the divert valve (0.3–0.4 min). Data files were calibrated post-run on the average spectrum from this time segment, using the Bruker HPC (high-precision calibration) algorithm. For ESI+ the capillary voltage was maintained at 4,200 V, the gas flow to the nebulizer was set to 2.4 bar, the drying temperature was 220°C, and the drying gas flow was 12.0 L min^−1^. Transfer optics (ion-funnel energies, quadrupole energy) were tuned on HT-2 toxin to minimize fragmentation. For ESI—the settings were the same, except that the capillary voltage was maintained at −2,500 V. Unless otherwise stated, ion-cooler settings were: transfer time 50 μs, radio frequency (RF) 55 V peak-to-peak (Vpp), and pre-pulse storage time 5 μs. After changing the polarity, the mass spectrometer needed to equilibrate the power supply temperature for 1 h to provide stable mass accuracy.

### Automated screening of fungal samples

Target Analysis 1.2 (Bruker Daltonics, Bremen, Germany), was used to process data-files, with the following typical settings: (A) retention time (if known) as ±1.2 min as broad, 0.8 min as medium, and 0.3 min as narrow range; (B) SigmaFit; 1,000 (broad) (isotope fit not used), 40 (medium), and 20 (narrow); and (C) mass accuracy of the peak assessed at 4 ppm (broad), 2.5 ppm (medium), and 1.5 ppm (narrow). Area cut-off was set to 3,000 counts as default, but was often adjusted for very concentrated or dilute samples. The software DataAnalysis (DA) from Bruker Daltonics was used for manual comparison of all extracted-ion chromatograms (EIC) generated by Target Analysis to the base peak chromatograms (BPC), to identify non-detected major peaks.

## Results

### Fungal isolates

**Figure 1:**
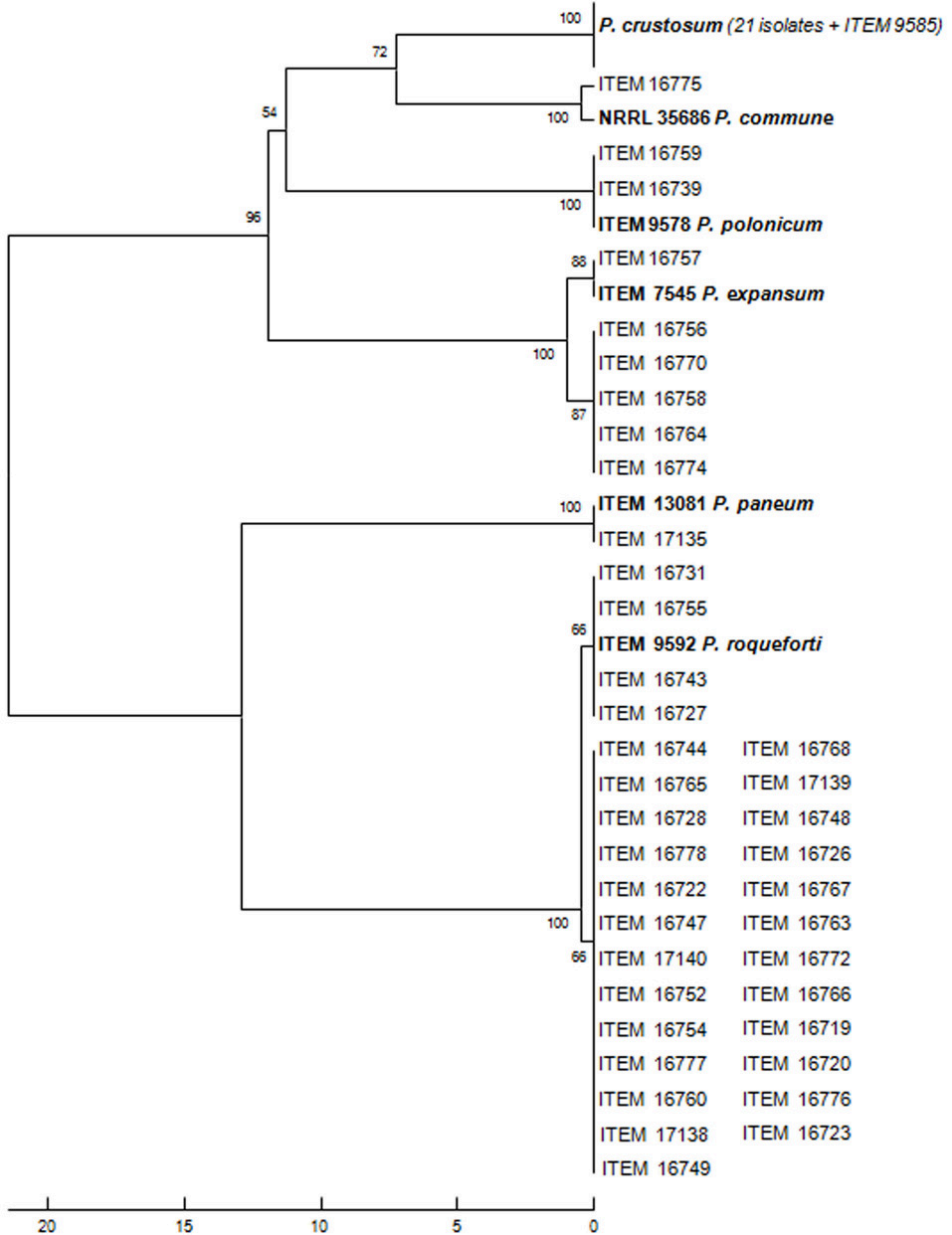
Evolutionary relationships of 60 isolates from olives and 6 species reference strains.

In the present study, molds were collected during fermentation from different lab- and industrial-scale table olive fermentations performed in Italy and Greece and from different Italian and Greek commercial products belonging to the four cultivars Leccino, Cellina di Nardò, Kalamàta, and Conservolea. A total of 60 fungal strains were isolated from DRBC medium plates. Molecular identification of all of those isolates was performed at species level by sequencing ITS and beta-tubulin gene. Species were identified using BLAST on the NCBI website (www.ncbi.nlm.nih.gov/BLAST/) and through comparison with the sequence database of *Penicillium* type strains sequenced at ISPA-CNR (Figure 1). The analysis revealed 21 *Penicillium crustosum* isolates from all the cultivars (>99% similarity to Acc. n. KJ410745.1), one *P. commune* isolate from Kalamàta (100% similarity to EF198566), six *P. expansum* from Cellina di Nardò and Kalamàta (>99% similarity to AY674400.1), one *P. paneum* (>99% similarity to AY674389.1), two *P. polonicum* isolates from Cellina di Nardò (>99% similarity to EU128563.1), 29 *P. roqueforti* isolates from all the cultivars (>99% similarity to AY674382.1).

### Characterization of fungi

**Figure 2:**
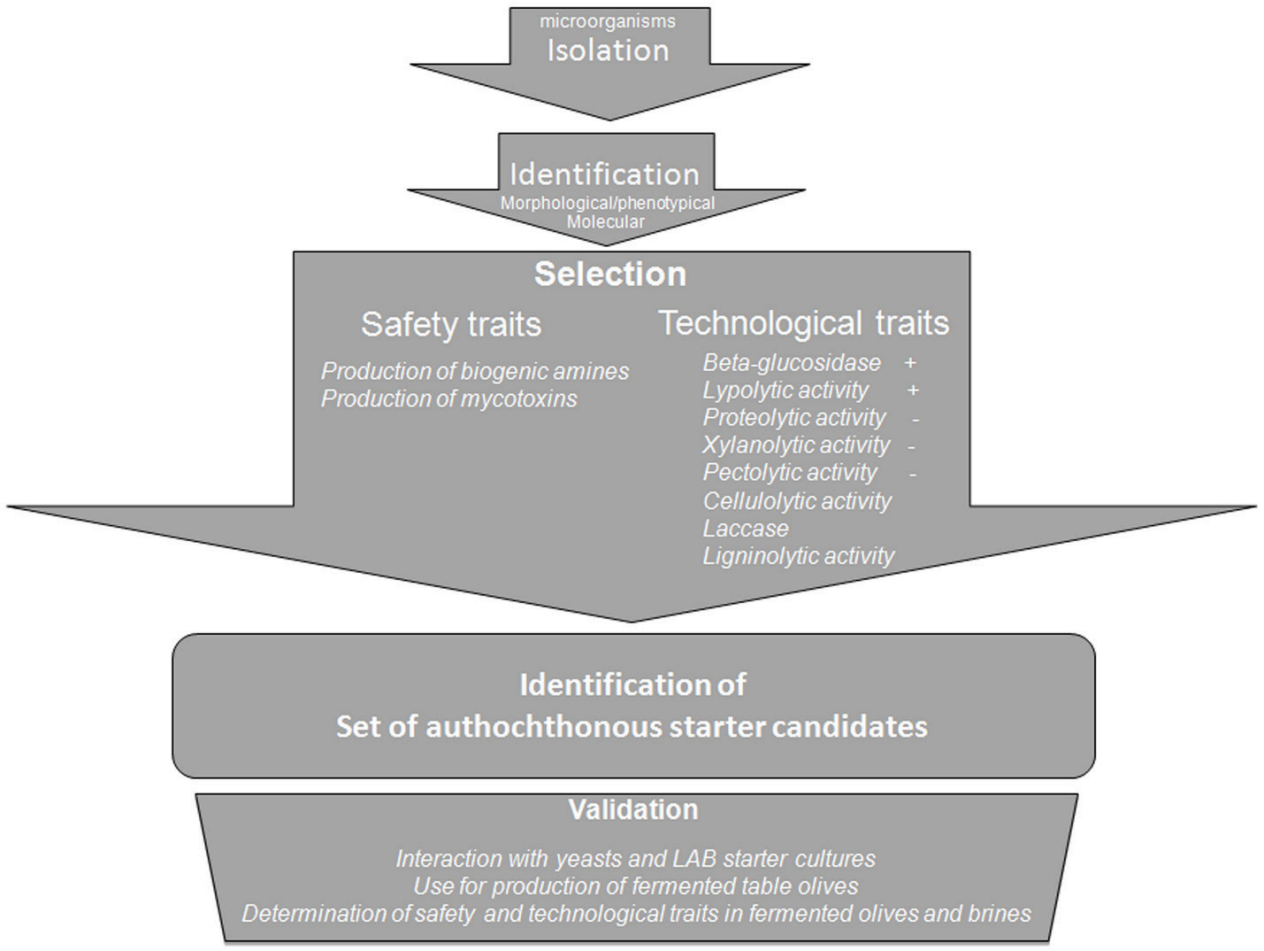
Flow-sheet for the selection of a mold starter culture.

A subgroup of 20 isolates (Table 1) was selected among the 60 fungal isolates, in order to perform biochemical characterization for their technological and safety traits (Figure 2). The subset included representative isolates for the different table olive cultivars, geographic localizations, morphotypes inside the same species. This subgroup consisted of 20 mold isolates in parallel subjected to:

1. Technological tests: (i) the presence of beta-glucosidase activity, required to degrade oleuropein and of lypolytic activity, involved in formation of several volatile compounds able to improve the flavor of olives; (ii) the absence of enzymatic activities (protease enzymes, cellulolytic, ligninolytic, pectolytic, and xylanolytic activities) with a possible negative effect on olive texture and quality.
2. Safety assessments for the production of biogenic amines and mycotoxins.

**Table 1:** Fungal isolates selected for biochemical characterization.

**ITEM**	**Sample**	**Species identification (beta tubulin)**	**Country**
16721	Leccino industrial fermentation 16	*Penicillium crustosum*	Italy
16750	Cellina di Nardò industrial fermentation 10		
17134	Cellina di Nardò commercial product		
16733	Cellina di Nardò industrial fermentation 4		
16720	Leccino industrial fermentation 16	*Penicillium roqueforti*	
16727	Leccino industrial fermentation 4		
16728	Leccino industrial fermentation 7		
16743	Cellina di Nardò industrial fermentation 13		
16744	Cellina di Nardò industrial fermentation 13		
16756	Cellina di Nardò industrial fermentation 10	*Penicillium expansum*	
17133	Cellina di Nardò commercial product	*Penicillium paneum*	
16775	Kalamata industrial fermentation	*Penicillium commune*	Greece
16741	Conservolea industrial fermentation A3 P2	*Penicillium crustosum*	
16753	Conservolea industrial fermentation A5 P2		
16762	Conservolea lab-scale fermentation 4		
16740	Kalamata industrial fermentation K3 P1		
17138	Kalamata lab-scale fermentation C	*Penicillium roqueforti*	
17139	Kalamata lab-scale fermentation C		
16754	Conservolea industrial fermentation A5 P2		
16763	Conservolea lab-scale fermentation 4		

Concerning technological characterization, specific qualitative plate tests were used to determine the presence of extracellular enzymatic activities (beta-glucosidase, lypolitic activities, cellulase, laccase, tyrosinase, xylanase, lignin modifying, pectolytic, and protease) in the 20 selected mold isolates.

All the isolates showed beta-glucosidase activity (Table 2). The isolate ITEM 16756 revealed the highest lipolytic activity (score 3), whereas ITEM 16728, 16733, 16740, 16741, 16750, 16753, 16754, 16762, 16763, 16775, 17138, 17139, and 17134 showed slight activity (score 1). No lipolytic activity was revealed in the remaining isolates. Although, no isolates produced degradation activities on milk agar, some isolate showed ability to degrade gelatine (ITEM 16720 with score 3; ITEM 16727, 16743, 16750, 16753, 16762, 16763, and 17139 with score 2; ITEM 16728, 16744, 17138, and 17135 with score 1; Table 2). Moreover, all tested isolates revealed the presence of different levels of pectolytic activity, whereas the ability to degrade xylan is absent in many of them (ITEM 16720, 16728, 16744, 16754, 16756, 16762, 16763, 16775, 17138, 17139, 17133; Table 2). Concerning lignin modifying enzymes, they were detected at low and medium levels only for the isolates ITEM 16721, 16741, 16756. Many isolates did not produce detectable cellulolytic activities (ITEM 16720, 16727, 16728, 16733, 16740, 16741, 16743, 16744, 16750, 16756, 16762, 17134, and 17133; Table 2). Laccase and tyrosinase activities were undetectable in all the tested molds (Table 2).

**Table 2:** Enzymatic activities of the selected molds isolated from table olives.

**ITEM**	**Beta glucosidase**	**Lypolytic activity**	**Cellulolytic activity**	**Laccase**	**Tyrosinase**	**Lignin modifying enzymes**	**Protease activity (Milk agar)**	**Protease activity (Gelatin)**	**Pectolytic activity**	**Xilanolytic activity**	**Aminoacid decarboxylation activities**			
											**Arginine**	**Phenylalanine**	**Tyrosine**	**Histidine**
16720	++	−	−	−	−	−	−	+++	++	−	−	−	−	−
16721	+++	−	++	−	−	+	−	−	++	++	++	−	−	−
16727	++	−	−	−	−	−	−	++	+	+	-	−	−	−
16728	++	+	−	−	−	−	−	+	+++	−	−	−	−	−
16733	+++	+	−	−	−	−	−	−	++	++	++	−	−	−
16740	+++	+	−	−	−	−	−	−	++	+++	++	−	−	−
16741	+++	+	−	−	−	+	−	−	++	++	++	−	−	−
16743	+	−	−	−	−	−	−	++	+	+	-	−	−	−
16744	++	−	−	−	−	−	−	+	++	−	-	−	−	−
16750	++	+	−	−	−	−	−	++	+++	++	++	−	−	−
16753	++	+	+	−	−	−	−	++	++	+++	++	−	−	−
16754	+	+	+	−	−	−	−	−	++	−	-	−	−	−
16756	+++	+++	−	−	−	++	−	−	+++	−	-	−	−	−
16762	++	+	−	−	−	−	−	++	++	−	++	−	+++	−
16763	+	+	+	−	−	−	−	++	++	−	-	−	−	−
16775	++	+	+	−	−	−	−	−	+++	−	+	−	−	−
17133	++	−	−	−	−	−	−	+	+++	−	-	−	−	−
17134	+++	+	−	−	−	−	−	−	++	+	++	−	−	−
17138	++	+	+	−	−	−	−	+	++	−	−	−	−	−
17139	++	+	+	−	−	−	−	++	++	−	−	−	−	−

For the determination of the amino acids decarboxylation activities, the value 0 was used to indicate isolates that remained white, isolates surrounded by an intense purple halo were marked with the value 3, isolates producing intense blue halo with the value 2, isolates producing slight blue halo with the value 1 (Table 2). None of the isolates were able to decarboxylate histidine, tyrosine and phenylalanine, precursors of histamine, tyramine and 2-phenyl-ethylamine, respectively. Only the isolate ITEM 16762 showed good decarboxylation activity (value 3) of tyrosine. Various levels of arginine decarboxylation activity were detected for the isolates ITEM 16721, 16733, 16740, 16741, 16750, 16753, 16762, 16775, 17134.

Fungal ability to produce secondary metabolites, included mycotoxins, was investigated by growing isolates in two different conditions: namely in CYA medium and CYA medium with the addition of the two constrains represented by salinity and acidity (5% NaCl and pH 5.5) (CYAS) in order to evaluate the role of salinity and acidity in affecting secondary metabolites production. The secondary metabolites were analyzed by the Target Analysis system (Klitgaard et al., 2014), able to screen each extract for 3,000 compounds, considering mass accuracy, isotope fit, and retention time (RT), producing a qualitative representation of all mycotoxins present in the sample (Table 3).

**Table 3:** Secondary metabolites produced in culture by molds isolates from table olives.

		**ITEM 16775**	**ITEM 16721**	**ITEM 16740**	**ITEM 16741**	**ITEM 17134**	**ITEM 16756**	**ITEM 17133**	**ITEM 16727**	**ITEM 16728**	**ITEM 16743**	**ITEM 16744**	**ITEM 16754**	**ITEM 17138**
		***P. commune***	***P. crustosum***	***P. crustosum***	***P. crustosum***	***P. crustosum***	***P. expansum***	***P. paneum***	***P. roqueforti***	***P. roqueforti***	***P. roqueforti***	***P. roqueforti***	***P. roqueforti***	***P. roqueforti***
Chaetoglobosin A	CYA						+							
	CYAS						+							
Chaetoglobosin C	CYA													
	CYAS						+							
Communesin A	CYA						+							
	CYAS						+							
Communesin B	CYA													
	CYAS						+							
Marcfortine	CYA							+						
	CYAS													
Cyclopiazonic acid	CYA	+												
	CYAS	+												
FKI-3389	CYA	+												
	CYAS													
Sclerotigenin	CYA	+												
	CYAS	+												
Anacine or similar compound	CYA		+			+								
	CYAS		+			+								
Andrastin A	CYA		+	+	+	+		+			+	+		
	CYAS		+	+	+	+		+			+	+		+
Cyclopenol	CYA		+	+	+	+								
	CYAS		+	+	+	+								
Dehydro cyclopeptin	CYA													
	CYAS		+	+	+	+								
Penitrem A	CYA		+	+	+	+								
	CYAS		+	+	+	+								
Roquefortine C	CYA		+	+	+	+	+		+	+			+	
	CYAS		+	+	+	+			+	+	+		+	+
Roquefortine X	CYA													+
	CYAS													
Viridicatol	CYA		+	+	+	+								
	CYAS		+	+	+	+								
Viridicatin	CYA													
	CYAS			+		+								
Mycophenolic acid	CYA								+	+	+	+	+	+
	CYAS								+	+	+	+	+	+
PR-toxin	CYA								+					+
	CYAS													
Terrestric acid	CYA													
	CYAS			+										
Agroclavine	CYA													
	CYAS										+	+	+	+

*^+^denotes the presence of a metabolite*.

Aflatoxins, Ochratoxin, and Patulin were not detected in any mold isolates. Secondary metabolites produced by the isolate ITEM 16728 (*P. roqueforti*) are not affected by the salt and acidity in the medium. At the two conditions (CYA and CYAS) it produced mycophenolic acid and roquefortine C.

The isolate ITEM 16775 produced cyclopiazonic acid and sclerotigenin in the two tested conditions, but FKL-3389 was released only in the medium without salt and acidic conditions (CYA). The isolate ITEM 16756 (*P. expansum*), in addition to chaetoglobosin A, communesin A, and roquefortine C, in presence of salt and acidity produced also chaetoglobosin C and communesin B. Among isolates belonging to the species *P. roqueforti*, all of them produced mycophenolic acid in absence of salt and acidity stress, whereas the isolates ITEM 16727 and 16754 produced roquefortine C in the two tested conditions. This last metabolite was revealed in ITEM 16743 and 17138 grown on salt and acidic medium. The PR-toxin producers ITEM 16727 and 17138 did not release this toxin in salt and acidic conditions. The isolates ITEM 16743, 16744, 16754, and 17138 also produced agroclavine in presence of salt and acidic pH (CYAS). Finally, the ITEM 17138 released roquefortine C and andrastatin A in salt and acidic conditions, whereas it produced roquefortine X in absence of these stresses.

Considering *P. crustosum* isolates, all of them produced dehydrocyclopeptin in stress (salt and acid) conditions in addition to andrastin A, cyclopenol, penitrem A, roquefortine C, viridicatol. In CYAS medium, in presence of the isolates ITEM 16721 and 17134 there anacin and viridicatin were also detected, whereas viridicatin was produced by ITEM 16740 and 17134 and terrestric acid was produced only by ITEM 16740.

Summarizing, the presence of salt and low pH affects in different way all metabolites: in particular they stimulate production of dehydrocyclopeptin and in some case also viridicatin in all *P. crustosum* (ITEM 16740 and 17134) strains tested and stimulated terrestric acid production in ITEM 16740; in *P. roqueforti* isolates ITEM 16743 and 17138 produced roquefortine C and only ITEM 17138 produced andrastin A, whereas a roquefortine derivative (present in ITEM 17138 grown in no salt and no acidic conditions) and PR-toxin (revealed in ITEM 17138 and ITEM 16727 in absence of salt and low pH conditions) were not detected; *P. expansum* ITEM 16756 produced chaetoglobosin C and communesin B but not roquefortine C; marcfortine was not detected in *P. paneum* ITEM 17133. *P. roqueforti* ITEM 16743, 16744, 16754, and 17138 produced agroclavine.

### Selection of fungi

Combining qualitative tests for both technological (the presence of desired enzymatic activities, i.e., beta-glucosidase and/or lipase activities, the reduced or absence of undesired traits, i.e., proteases, pectolytic and/or xylanolytic activities) and safety assessments of tested fungal isolates, it was possible to select some interesting isolates that can be considered as putative candidates for future studies as co-starters with yeasts and lactic acid bacteria. At the end of this procedure, the set of candidate strains for standardized fermentation of table olives includes: *P. roqueforti* ITEM 16728 selected from Leccino cultivar, *P. paneum* ITEM 17133 and *P. roqueforti* ITEM 16744 selected from Cellina di Nardò cultivar, *P. roqueforti* ITEM 17138 selected from Kalàmata cultivar and *P. roqueforti* ITEM 16754 selected from Conservolea cultivar. It is to be considered, however, that table olives fermentations need to be performed in real conditions using the selected candidate strains in order to evaluate both safety and technological traits in olive and brine samples.

## Discussion

Molds belonging to *Penicillium* and other molds genera (*Aureobasidium, Aspergillus, Geotrichum*) are often associated to Black “Greek style” table olives. Their role in table olives production is not completely understood yet. At present, they are considered as contaminating microorganisms, responsible for spoilage (texture softening, production of moldy odor and taste) and potentially toxicity (Heperkan, 2013).

The interest on molds associated to table olives starts from the consideration that they are used as secondary starter cultures in Europe to process meat and cheese products, affecting positively their flavor, taste, texture, offering protection against spontaneous undesired microorganisms, delay of rancidity, stabilization of color, oxygen, and light protection, etc. On the other end, they can also release highly toxic secondary metabolites such as mycotoxins. Non-toxigenic and technological mold starters can be exploited for standardized fermentation, hindering the growth of undesired microorganisms, similarly to what have been done for dry-cured meat using *P. nalgiovense, P. chrysogenum*, and recently of *P. salamii* (Sunesen and Stahnke, 2003; Delgado et al., 2016; Magistà et al., 2016); for the production of white cheeses using *P. camemberti* (Brie and Camembert), and for the production of blue cheeses (Roquefort, Gorgonzola, Stilton, Gammelost, etc.) using *P. roqueforti* (Geisen, 1993).

In the present paper, for the first time we (i) identified and characterized mold isolates associated to four different black table olives cultivars and (ii) selected some of them for their potential use as co-starter for table olives production. It can be recommended to use species from series *Roquefortorum* (*P. roqueforti* and *P. paneum*) in olive fermentation, because these are the only Penicillia that can tolerate higher concentrations of acetic acid and lactic acid produced by the lactic acid bacteria (Frisvad et al., 2004; Houbraken et al., 2010). In other lactic acid and acetic acid containing foods and feeds *Roquefortorum* species are also the dominating Penicillia for example cocoa, silage, rye bread, sauerkraut (O'Brien et al., 2006; Copetti et al., 2011; Gallo et al., 2015) etc., as long as the concentration of acetic acid is sufficiently high. If the concentration of these acids is low, toxigenic species such as *P. crustosum, P. commune*, and *P. expansum* may thrive. These toxigenic fungi were found in olives in this study, so it is recommended to secure that sufficiently high concentration of acetic acid and lactic acid is present in the table olives.

For the first time, a flow sheet has been proposed for selection of molds associated to table olives. After identification using morphology/phenotypic characteristics and molecular approaches, the mold isolates can be selected using two main criteria (Figure 2): presence/absence of enzymatic activities and of toxic compounds. This selection step can help to individuate candidates to be used in further validation step consisting in the study of their positive/negative interaction with already existing starter cultures for table olives and in the use of them alone or in combination with yeasts and LAB for table olive production.

Molds associated to Leccino, Cellina di Nardó, Kalamàta, and Conservolea fermented table olives were for the first time isolated and identified. Classical morphological identification combined with molecular approach was used to improve resolution and discriminating power inside *Penicillium* species (Baffi et al., 2012). The *P. commune, P. crustosum, P. expansum, P. roqueforti* were already reported in previous works as associated to table olives (Ghitakou et al., 2006; Heperkan et al., 2006; Baffi et al., 2012), whereas the species *P. paneum* and *P. polonicum* were not reported earlier in this product and they could represent an occasional contamination, considering the low number of colonies observed. Following the selection strategy proposed for yeasts and lactic acid bacteria, both technological and safety tests have been chosen in order to perform a selection of mold isolates.

A subgroup (20) of them, selected in order to represent the overall observed molecular biodiversity, was assayed for their positive trait to produce beta-glucosidase, responsible of polyphenols degradation (in particular oleuropein) and debittering of table olives together with impact on flavor, due to the production of secondary metabolites (Bevilacqua et al., 2013). Seventeen isolates showed good beta-glucosidase activity, representing promising candidates able to help other starters to degrade oleuropein, reducing time for debittering and NaCl content in table olive processing. Several isolates (14) revealed the presence of lipase activity, desired activity that could improve the aromatic profile of fermented olives (Savitha et al., 2007; Rodríguez-Gómez et al., 2012).

In this preliminary study, following the approach previously used for yeasts and LAB (Arroyo-López et al., 2012; Bevilacqua et al., 2013; Bleve et al., 2014, 2015), the presence of proteolytic activity was considered as a negative characteristic, since it is responsible for the release of aminoacids and ammoniacal nitrogen causing in turn a pH increase and the risk for the product to be unsafe (Tosi et al., 2008; Ledenbach and Marshall, 2009). Moreover, proteolytic activity could be also responsible of undesired impact on olive quality provoking olive softening. Although, no isolates produced detectable activity on milk agar, 12 of them metabolized gelatin at different levels (score 1–3). However, considering microbiological research performed so far and scientific data available, it is not possible to definitely establish the real impact of these enzymatic activities on table olives quality. In fact, the relative influence of fungal enzymatic activities could contribute to the maturation of aroma together with other parameters, such as raw materials and processing conditions (Meynier et al., 1999; Harkouss et al., 2012). Other enzymatic activities that have been described as undesired traits in yeasts, such as cellulases, xylanases, pectinases, need to be verified for molds when they are inoculated in table olives. Also in this case, scarce information is still available about the compounds and enzymes produced by the mold population during fermentation and their concentrations in packed olives. This is an important aspect since the presence of molds that are able to secrete these enzymes in table olives could modify in a not predictable manner the nutritional composition of table olives and of their organoleptic characteristics.

It is nevertheless worth noting that beta-glucosidase, cellulase, xylanase, pectinase, lignin modifying, lypolytic, and proteolityc enzymatic activities detected in many mold isolates can be of interest also for major industrial applications. Potential biotechnological uses of Penicillia, isolated from fresh olive fruits, olive paste and pomace, were suggested by Baffi et al. (2012). Analogously to the approach followed for yeasts (Bleve et al., 2011), mold isolates (as whole cells in free or immobilized forms) or enzymes purified from different mold species can be useful for treatment and/or bioconversion of agro-food by-products, such as wastewaters deriving from olive oil and table olives industry.

Table olives are one of the most contaminated fermented food by biogenic amines like putrescine, cadaverine, and tyramine, as reported in “Zapatera” spoiled olives (Hornero-Mendez and Garrido-Fernandez, 1994) and in naturally fermented (Greek-style) table olives (Tofalo et al., 2012).

Recently, in addition to LAB, also yeasts have been considered as a source of biogenic amines and preliminary assays have been included in selection programs to check their ability to produce these compounds “*in vitro*” conditions (Bevilacqua et al., 2013; Bleve et al., 2014, 2015). In order to reduce the risks for consumer, these tests have been introduced in this study also for molds, to select microbial sources unable to produce biogenic amines in fermented table olives.

In this paper a preliminary screening of mold isolates, on media with or without salt and acidic constraints, was also carried out for qualitative analysis of many secondary metabolites (including mycotoxins) in an attempt to identify isolates potentially low producer of mycotoxins.

The three regulated mycotoxins Aflatoxins, Ochratoxin A, and Patulin were not detected in any mold strains isolated from the fermented black olives.

*P. expansum* ITEM 16756 produced chaetoglobosin A and communesin A independently from the presence of absence of salt and low pH, whereas communesin B was revealed only in presence of these conditions. The isolation of chaetoglobosin A, characterized by embryolethal but not teratogenic effects on chickens was reported in the genus *Penicillium* (Veselý and Jelínek, 1995). Besides patulin and roquefortine C, Andersen et al. (2004) described the presence of chaetoglobosin A and communesin B in *P. expansum* contaminated fruit juices and potato pulp. Oral toxicity in cockerels, embryonic chickens, rats, and mice, cytotoxic activity in HeLa cells, teratogenic activity in mice were demonstrated for chaetoglobosin A (Sekita et al., 1982; Cherton et al., 1994), whereas communesin B is cytotoxic to lymphocytic leukemia cells (Numata et al., 1993). However, despite their negative effects reported in literature, these mycotoxins are not regulated as food contaminants.

The presence of cyclopiazonic acid (CPA), an indol–tetramic acid mycotoxin produced by the nearly ubiquitous molds *Aspergillus* and *Penicillium*, was revealed in *P. commune* ITEM 16775.

Although, CPA is not considered to be a potent acute toxin, it has different target organs (hepatic tissue, and spleen). Its toxicity and symptoms are linked to its ability to alter normal intracellular calcium flux and potential immunomodulatory effect in “*in vitro*” studies. Several *Penicillium* species (*P. camamberti, P. cyclopium, P. viridictum, P. griseofulvum, P. crustosum*, and others) associated to cheese (Camembert, brie, cheddar, cream, cheese rind, etc.), acorn, barley, corn, peanuts, ham, and chicken meat were reported as producers of CPA (Burdock and Flamm, 2000).

The potent neurotoxin penitrem A has been detected in important agricultural commodities (maize, rice, wheat, oat, rye, barley, and sorghum) and in moldy cream cheese and it is produced by several species of fungi. According to the results reported by El-Banna and Leistner (1988), also all isolates of *P. crustosum* tested in this study were able to produce detectable levels of penitrem A.

The *P. expansum* ITEM 16756 and all isolates of *P. crustosum* and of *P. roqueforti*, with the exception of the isolate ITEM 16744, produced roquefortine C. This mycotoxin has neurotoxic properties (Wagener et al., 1980), but the data on its neurotoxicity are equivocal (Fog Nielsen et al., 2006). Several studies have revealed the presence of roquefortine C in blue-veined cheeses (Ohmomo et al., 1975; Scott and Kennedy, 1976; López-Díaz et al., 1996). It is always found in cheeses, since fungal strains (*P. camamberti* and *P. roqueforti*) used as starters in the dairy industry are able to produce this toxin *in vitro*. However, the relatively low toxicity and low concentrations of this toxin in blue cheese make this product safe to be consumed (Finoli et al., 2001).

According to data reported in this paper and by other authors (Frisvad et al., 2004), *P. roqueforti* is also known to produce mycophenolic acid (MPA) and PR-toxin (detected only in ITEM 16727 and 17138).

Although, MPA possess immunosuppressive effects (Bentley, 2000), compared to other mycotoxins (T-2 toxin, gliotoxin, DON, and patulin), roquefortine C and MPA showed low acute cytotoxicity on the human intestinal cell line Caco-2 (Rasmussen et al., 2010).

PR-toxin is instead the most toxic metabolite produced by *P. roqueforti* because of a high toxic or lethal effects in rats, mice, and cats and is mutagenic in the Ames test (Arnold et al., 1978; Fog Nielsen et al., 2006). However, PR-toxin is unstable because it quickly reacts with different salts, amino acids (especially Sulfur containing amino acids), amines, casein, and the decomposition products resulting in the less toxic PR imine (Erdogan et al., 2003).

Despite the detection of variable contents roquefortine C and MPA in several blue-veined cheeses, no cases of intoxications linked to the consumption of these products have ever been reported (Fontaine et al., 2015). Roquefortine C and MPA combined exposure was studied on human intestinal cells (Caco-2 cells) and on monocytes (cancer lineage model cell THP-1) by Fontaine et al. (2015). These authors demonstrated that roquefortine C and MPA may not create problems of acute exposure in food, since human are exposed at relatively low levels and these toxins show low acute cytotoxic effects in comparison to other regulated mycotoxins. Agroclavine produced by *P. roqueforti* is the precursor of isofumigaclavine A, and its hydrolysis product, isofumigaclavine B, are identical to Roquefortine A and B, respectively. Low levels or traces of Isofumigaclavine A and B were found in marbled and blue cheese. The toxicological effects of these substances are not well-known, but some neurotoxic data are described by several studies (Ohmomo et al., 1975; Scott and Kennedy, 1976; Scott, 1984). However, Agroclavine itself is probably not very toxic, although there are few available data on its toxicity.

Although, several isolates of *P. expansum* were shown to be able to produce patulin and citrinin (Andersen et al., 2004), and different isolates of *P. paneum* were responsible of production of patulin (O'Brien et al., 2006), none of these toxins were detectable for all the tested isolates.

Considering technological and safety data collected for all tested isolates, at the end of this procedure, it is possible to select some isolate, autochthonous for each table olive cultivar, suitable for future validation tests as co-starters with yeasts and lactic acid bacteria and for production of fermented table olives. Tests on an *ad hoc* optimized “olive agar” and then directly on fermented table olives and brine samples inoculated with selected molds might indicate the quantity and which biogenic amines and mycotoxins will be eventually produced on the final product during a fermentation process in real conditions.

## Author contributions

Fundamental contributions to the conception and design of the work, acquisition, analysis and interpretation of data: GB, SB, AS, AL; Acquisition, analysis, elaboration, and interpretation of data: SB, AS, GB, JF, MT, AC; Drafting the work and revising it critically for intellectual content: GB, AS, GP, GM, AL. All authors approved the final version of the manuscript to be submitted for publication and agreed to be accountable for all aspects of the work in ensuring that questions related to the accuracy and integrity of any part of the work are appropriately investigated and resolved.

### Conflict of interest statement

The authors declare that the research was conducted in the absence of any commercial or financial relationships that could be construed as a potential conflict of interest.
